# The family caregiver-targeted web-based intervention “narstaende.se” facilitated everyday life for couples facing life-threatening illness: A qualitative study

**DOI:** 10.1177/02692163251327893

**Published:** 2025-04-12

**Authors:** Cecilia Bauman, Viktoria Wallin, Sandra Doveson, Per Fürst, Peter Hudson, Ulrika Kreicbergs, Anette Alvariza

**Affiliations:** 1 Department of Health Care Sciences, Marie Cederschiöld University, Stockholm, Sweden; 2 Department of Nursing Science, Sophiahemmet University, Stockholm, Sweden; 3 Department of Neurobiology, Care Sciences and Society (NVS), Karolinska Institute, Stockholm, Sweden; 4 Research and Development Unit/Palliative Care, Stockholms Sjukhem, Stockholm, Sweden; 5 Centre for Palliative Care St Vincents & The University of Melbourne, Melbourne, VIC, Australia; 6 End of Life Research Department, Vrije University, Brussels, Belgium; 7 Great Ormond Street Institute of Child Health, University College London, London, UK

**Keywords:** Palliative care, family caregivers, intervention, web-based, internet-based intervention, home care, psychoeducational

## Abstract

**Background:**

Life-threatening illness affects both patients and spouses, and spousal caregivers report high levels of distress. Web-based interventions could benefit spouses’ and patients’ needs and shared everyday life.

**Aim:**

To explore how a family caregiver-targeted web-based psychoeducational intervention influences couples’ experiences of sharing everyday life at home while facing life-threatening illness.

**Design:**

This qualitative sub-study involved dyadic interviews with couples (spouse-patient) where the spouse was allocated to the intervention arm of a randomized controlled trial evaluating a web-based family caregiver-targeted intervention. Data were analyzed using Interpretive description.

**Setting/Participants:**

Participants were recruited from five specialized home care services in Sweden. In total, 32 participants, spouses (n = 16) and patients (n = 16) were interviewed as couples after the spouse had accessed the intervention for 4 weeks.

**Results:**

Couples described how the spouses’ access to the intervention had provided knowledge that enhanced the couple’s understanding of each other’s strategies for managing the impacts of the illness. The topics covered in the intervention prompted the spouses to initiate conversations that helped couples maintain a sense of mutuality. The intervention provided support to balance the tension between previous and new relational roles, which had changed due to the patient’s illness.

**Conclusions:**

Altogether, the results show that the benefits of family caregiver-targeted interventions may extend from spouse to patient, facilitating their everyday life. Our findings complement previous intervention evaluations by providing insights into how they may be effective. The goal should be that interventions potentially benefit patients and family caregivers.

**Trial registry:**

The randomized controlled trial is registered at ClinicalTrials.gov, ID NCT05785494.

**What is already known about the topic?**

- Care and support are often provided by a patient’s spouse.
- Illness affects the well-being and everyday life of patients and spouses, both individually and as a couple.

**What this paper adds**

- The web-based intervention provided knowledge that enhanced couples’ understanding of each other’s strategies for managing the impacts of the illness.
- The web-based intervention initiated conversations that helped couples maintain a sense of mutuality.
- The web-based intervention provided support to balance the tension between previous and new relational roles.

**Implications for practice, theory, or policy**

- Family caregiver-targeted web-based interventions may facilitate spouses’ and patients’ experience of sharing everyday life.
- Findings may guide future intervention development through novel insights into how the benefits of a web-based intervention extend from spouse to patient, helping them to manage illness as a couple.
- The present study adds further dimensions and knowledge to family caregiver intervention evaluation by also including patient perspectives.

## Background

The care and support of a patient facing a life-threatening illness are often provided by the patient’s spouse.^1^ Illness affects both patients and spouses, individually and as couples because their psychological well-being is interrelated and shared.^2–4^ Many spouses naturally assume a caregiver role at the end of their partner’s life,^5^ yet, the caregiver role can negatively affect their physical and emotional health and well-being.^6–10^ Patients may also feel like a burden to their family, which can create an imbalance that affects the couple’s relationship.^11^ Home-based palliative care often requires adjustments in the home environment and daily routines, which can negatively impact couples’ everyday lives.^12^ Spousal caregivers report higher levels of distress and depressive symptoms than other family caregivers.^1,6^ and often have unmet needs for information and psychological support.^13,14^ However, spousal caregivers may be reluctant to seek help for their needs^15^ and may therefore not access support that is delivered in person.^16^

Web-based psychoeducational interventions, despite variations in format, design, and aim, have become more prevalent. These interventions may provide available support for family caregivers,^17–19^ reduce depression, anxiety,^20^ and emotional distress^20,21^ and address diverse needs of family caregivers over time.^22^ Learning from the outcomes of such interventions may inform palliative care practices and policies.^23^ However, it remains unclear how these interventions could influence the everyday lives of spouses and patients facing life-threatening illness, particularly, how benefits might extend from spouse to patient and affect them as a couple.

## Aim

To explore how a family caregiver-targeted web-based psychoeducational intervention can influence couples’ experiences of sharing everyday life at home while facing life-threatening illness.

## Methods

### Design

The present study used an exploratory design with a qualitative inductive approach.^24^ It follows the Medical Research Council guidelines for complex intervention research,^25^ and is a sub-study of a randomized controlled intervention trial (NCT05785494) evaluating a web-based psychoeducational intervention for family caregivers. The intervention aimed to promote family caregivers’ preparedness for both caregiving and for death.^26^ Data for the present study were derived from dyadic interviews with couples. Spouses in the intervention arm of the randomized controlled trial had access to the intervention, whereas patients did not.

### The intervention

A web-based psychoeducational intervention is provided on the website “narstaende.se,” specifically targeting family caregivers. The intervention was co-developed by healthcare researchers, clinicians, information systems researchers, digital communication strategists, and IT consultants. Feasibility testing demonstrated both acceptability and usability.^27^ The content is based on empirical research^28–30^ and Andershed and Ternestedt’s^31^ theory on family caregivers’ involvement in care. According to this theory, involvement in care can be experienced as either “in the light,” where family caregivers feel informed and acknowledged, or “in the dark,” where they feel isolated and uninformed. It highlights three principal needs that shape involvement: *knowing* (knowledge of symptoms and diagnosis), *being* (shared time), and *doing* (practical tasks).

The intervention provides 23 short videos, between 2.5 and 8.5 min long, with descriptive titles to allow family caregivers to select preferred topics. The content is organized into three main domains: “Support for you – being a family caregiver,” “How to give support,” and “Talk about it” (Table 1). The videos are supplemented by informative texts, web links, and a chat forum moderated by the research group.

**Table 1.** The domains that are addressed on the intervention website.

Content	Support for you – being a family caregiver	How to give support	Talk about it
Video titles	Many different emotions	Nutrition and weight	Nice to have talked about
	Meeting others	Appetite loss	Talk about dying
	Ask for help	Pain relief	The loss
	Finding energy	Little helpful things	Withdrawal of treatment
	Continue working or not	Fatigue and rest	Thoughts when terminating active illness treatment
	Concerns about the future	Being a family member and a family caregiver	When death is closing in
	May I be happy?	Relieve the bad and find the good	Funeral/memorial ceremonies
	Being me		
	Breathing exercise		
Text titles	To ask for help	Respiratory distress	Funeral/memorial ceremony
	Living with a seriously ill person	Loss of weight and appetite	Death and dying
	Children and young people as close relatives	Constipation	Family member – family caregiver
	As death is closing in	Assistive devices	Society’s support system, financial/legal aspects
	After death	Nausea	
	Grief	Worry, anxiety, sadness	
		Advice on how to assist someone with personal hygiene	
		Pain	
		Tiredness – fatigue	

### Setting

The present study is part of a randomized controlled trial that included family caregivers from five specialized home care services in a metropolitan area in Sweden. Services included in the randomized controlled trial comprise multidisciplinary teams and provide 24/7 home care. A majority of patients have complex palliative care needs and limited survival expectancy, regardless of diagnosis, although most are diagnosed with advanced cancer. Each service unit manager received written and verbal information about the study and approved the recruitment of eligible participants through patient medical records by three researchers with clinical employment.

#### Participants and inclusion criteria

The randomized controlled trial included family caregivers involved in the care of a patient (both ⩾18 years) with a life-threatening illness and palliative care needs receiving specialized home care. Family caregivers had to communicate in Swedish to be eligible. The trial included 205 family caregivers, with half allocated to the intervention arm and given access to the web-based intervention. The present sub-study recruited participants from the intervention arm. Only couples, in which one was the spousal caregiver and the other a patient, were included.

#### Sampling and recruitment for the present study

**Figure 1.**
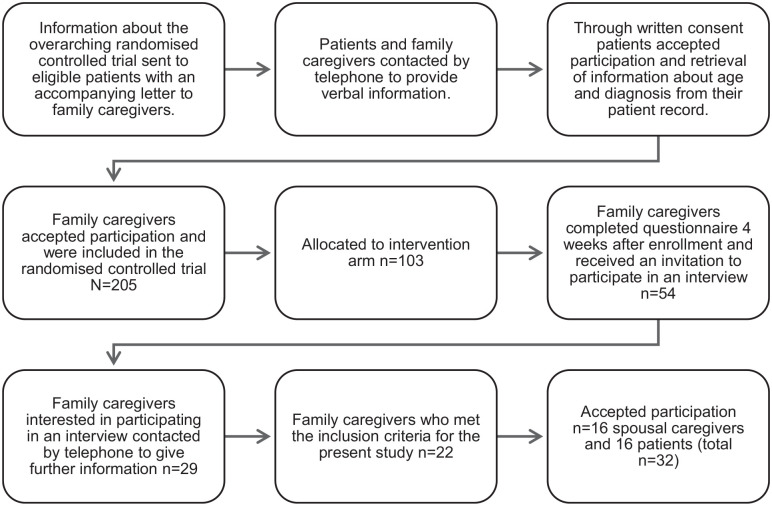
The inclusion procedure.

After 4 weeks of having access to the intervention, family caregivers were asked to complete a questionnaire including an invitation to participate in an interview. Spouses who accepted the invitation were contacted by telephone, given verbal information, and then informed the patient, and obtained their verbal consent. Written consent was obtained from both spouses and patients before the interview commenced. The inclusion procedure is described in Figure 1. Sampling was consecutive and the interviewer had no prior relationship with the participants. Sample size was continuously assessed based on information power, which considers the richness of data in relation to the study aim.^32^

### Data collection

Data for the present study were generated between January and December 2023 through semi-structured dyadic interviews conducted by the first author with spouses and patients together as couples. Dyadic interviews allow for in-depth narratives formed through interaction and co-creation between the interviewees.^33^ Based on participants’ preferences, 13 interviews were conducted face-to-face (12 in the couples’ homes and 1 in facilities provided by the interviewer), 2 via videoconferencing software, and 1 via telephone. A study-specific interview guide with open-ended questions covering four main areas was used (Table 2). It addressed the main domains of the intervention, focusing on implications for the couples’ everyday lives. All interviews were audio-recorded, and field notes of contextual information were taken. The interviews lasted between 25 and 89 min.

**Table 2.** Examples of areas and questions covered in the interview guide.

Expectations and reasons for participating	Support received from the intervention	Communication	Mutual support
Could you tell me about your [the patient’s] thoughts when [the spouse] was invited to take part in this website? What were your [the spouse] expectations before taking part in the website? Could you describe how you both discussed potential participation?	What has it been like for you [the patient] to know that your spouse has access to the website? How has this been a support to you? What did your spouse share with you about what is on the website?	Could you tell me about what influence the website has had on the way the two of you talk about things you perceive as difficult? How has the website been a support for you [the spouse] in talking to your partner?	Could you tell me about how you support each other now that [the patient] is ill? How has the website been helpful for you to support each other? What importance has the website had for you in your daily life?

### Data analysis

Data were analyzed using Interpretive description, a qualitative approach with epistemological roots in nursing.^24,34^ This analytic framework was chosen since this study seeks to understand complex and dynamic issues, possibly facilitated by interpretation informed by clinical experience.^35^ The authors have extensive knowledge and experience in palliative care, as clinicians (5 female nurses, 1 male nurse, and 1 male physician) and researchers, which informed data interpretation.^34,36^ Audio recordings were transcribed verbatim, including non-verbal expressions. Data transcripts were read several times to gain a sense of the whole. The first author created lengthy data excerpts pertaining to the study aim^24^ preserving the couples’ dialog and contextual understanding for further analysis. During careful discussions among the authors, the excerpts were organized into broad tentative themes, which developed throughout the analysis process.^34^ Each excerpt was given a description of its content to interpret the data and develop themes. The analysis remained open to divergent cases to enrich the findings. The findings were not validated by the participants.

### Ethical considerations

The study adheres to the Declaration of Helsinki.^37^ Ethical approval was obtained from the Swedish Ethical Review Authority on April 23, 2022 (approval number 2022-02218-02) and on December 14, 2022 (approval number 2022-06623-02). Participants were informed of their right to withdraw from the study and that their identity would be protected to ensure confidentiality. All participants provided verbal and written consent.

## Results

In total, dyadic interviews were conducted with 32 participants, 16 spouses and 16 patients (Table 3). The findings are organized into three themes: *Enhances understanding through new knowledge*, *Initiates conversations that maintain mutuality*, and *Provides* s*upport to balance the tension between previous and new relational roles.*

**Table 3.** Participant characteristics.

C﻿haracteristics	Spouses (n = 16)	Patients (n = 16)
Age, median (IQR), range	72.5 (15.5), 46–85	75 (7.5), 46–85
Gender		
Male	6	10
Female	10	6
Employment status		
Employed	6	0
On sick leave	0	3
Retired	10	13
Diagnosis		
Cancer		13
Advanced lung disease		2
Heart failure		1
Duration of incurable illness, in years, median (IQR), range		1.5 (3), 0–13

### Enhances understanding through new knowledge

Couples referred to the intervention as a resource where the spouse could gain knowledge about the illness and its implications for their everyday life, particularly through the domains of being a family caregiver and how to give support. Some of the new knowledge spouses gained was transferred to the patient, which ultimately benefited them as a couple. This knowledge helped couples understand aspects of their shared everyday life that they had not previously considered, such as gaining a better understanding of each other’s strategies for managing the impacts of the illness. This helped them come together to facilitate the management of challenges in their shared everyday life caused by the illness. Couples emphasized that the need for a source of knowledge, such as the intervention, depended on the impacts of the illness they were currently facing and on personal preferences, as described by a couple:

*Spouse: “I use [the intervention website] to understand and process. I’m one of those people who wants to be well informed, about [the patient’s] symptoms and illness.”*

*Patient: “Yes, and I think you know more about it than I do.” (Couple 9.* Husband, 58 years old, caring for his 60-year-old wife)

Spouses conveyed that the intervention provided them with knowledge about the extent to which the illness affected the patient, enabling them to understand why the patient sometimes behaved differently now in everyday situations compared to before the illness. Knowing that changes in behavior could occur because of the illness prompted spouses to adjust their reactions to the patient. This made the experience of sharing everyday life easier, as a spouse reported:

“*I’m a little more understanding [after using the intervention website]. Before [the intervention], I could get a bit irritated if he fell asleep when we were [watching TV]. Not like when he was well and used to be a night owl. Now I let him sleep. I’m more understanding [from the intervention website] that his body might be tired.” (Couple 15.* Wife, 79 years old, caring for her 85-year-old husband)

Even though patients did not have access to the intervention, they described that their spouse had access provided them knowledge about that the illness also affected their spouse. This broadened the patient’s understanding of their spouses’ point of view since they had typically perceived their shared everyday life from their own perspective and had therefore not fully understood their spouse’s experience. However, couples also pointed out that the intervention’s exclusive targeting of family caregivers could limit its influence on them as a couple. Spouses expressed that it would have been helpful if also the patient had access to the intervention, as it could have provided knowledge for both of them, helping them better understand how their individual approaches to the illness affected each other:

*Spouse: “[The intervention] could also be important for the person who is ill. A website like this could help us both understand things better. So, it’s really for you [the patient] as well.”*

*Patient: “Yes, I may not always understand your situation or our kids’ situation. So, it’s obvious, really, that it could be important for me as well.” (Couple 6.* Wife, 59 years old, caring for her 64-year-old husband)

### Initiates conversations that maintain mutuality

Spouses had initiated conversations with the patients as a result of their participation in the intervention. Couples explained how the spouse had been helped by the topics addressed on the intervention website, which provided a starting point for conversations. This was described by the couples as an opportunity to express their feelings and experiences to each other, as described by a couple:

*Spouse: “The fact that I’m processing things myself [through the intervention] makes it easier to bring them up and talk about them. Just having that ‘food for thought’ makes you more aware, so it makes it easier to talk about things.”*

*Patient: “Yes, and I think we’re pretty open about things?”*

*Spouse: “We are. I feel like I can tell people now, I feel like I should tell people how I’m doing.” (Couple 3.* Wife, 73 years old, caring for her 73-year-old husband)

Spouses described how their participation in the intervention had helped them as couples by creating a mutual sense of permission and comfort to discuss certain topics. The intervention was described as serving as a reminder of the limited time the couples had together, a topic the spouses felt was important but uncomfortable to discuss with the patient. Further, it allowed the spouse to bring up topics that had previously felt forbidden or challenging, providing a way to approach them without having to reveal their feelings. This was expressed by a patient who reflected on the importance of the intervention for his spouse:

“*[The intervention website] could be like a basis for conversation. Maybe it’s easier to ask things after watching these videos. Then you could say, ‘This is what it said on the website, how do you feel about it?’ instead of having to say what you think yourself.” (Couple 6.* Wife, 59 years old, caring for her 64-year-old husband)

Spouses described feeling like the “bad guy” in the couple because of their reactions to the patient’s change in behavior due to the illness. The intervention had given spouses the courage to discuss this with the patient.

Couples regarding themselves as open with each other found that the web-based intervention had encouraged them to maintain open communication. Spouses described how they began to reflect on things while using the website, which prompted communication with the patient. Patients described how this helped them understand that their spouse might feel the need to talk things through, even if they did not feel the same way, thus promoting mutuality.

### Provides support to balance the tension between previous and new relational roles

Couples expressed a desire to restore balance in their relationship, which became increasingly challenging as illness dominated their shared everyday life. They described the spouse’s access to the intervention as valuable for managing and enduring the strain caused by the illness, supporting them in balancing their previous roles with the new ones they had assumed, especially as these roles increasingly revolved around the patient. The patients recognized the challenges their spouse faced in balancing dual roles as caregiver and spouse. They expressed gratitude for their spouse’s efforts and hoped the intervention would provide support to them. They also believed that the intervention increased the likelihood that they both would have their support needs met, as a patient said:

*“We both need support, although different kinds of support, [we have] different needs to fulfill. So, I’ve felt like [the intervention] is a good thing, because the more help [the spouse] can get, the better.” (Couple 9.* Husband, 58 years old, caring for his 60-year-old wife)

Patients also expressed hope that the intervention would reassure their spouses that they were not facing their challenges alone. They were grateful for the support their spouse received through the intervention since they felt unable to provide this themselves. Patients acknowledged that a lot of attention was focused on them because of their illness, which they perceived could potentially overshadow their spouse’s needs. Knowing that the spouse had access to support through the intervention helped restore balance by addressing the uneven focus and ensuring that both their needs were recognized, as described by a couple:

*Patient: “It’s great [that the spouse has access to the intervention]. I don’t think you would disagree with that?”*

*Spouse: “No, and I think you’ve understood that I sometimes find things challenging.”*

*Patient: “Yes, that’s exactly why [the intervention] is so important.” (Couple 3.* Wife, 73 years old, caring for her 73-year-old husband)

Spouses conveyed that the intervention allowed them to gradually come to terms with the impact of the illness, rather than being abruptly confronted with the changing relationship as the patient’s illness progressed. A spouse said:

“*There are experiences [shown on the intervention website] of situations that others have been through that I haven’t. You can either go through it the hard way, or you can be somewhat prepared, even if it’s difficult. Until you’ve been in these situations, you may not realize what it’s all about.” (Couple 13.* Husband, 75 years old, caring for his 76-year-old wife)

Patients also recognized the importance of the intervention for their spouse at this time, as it could potentially help the spouse manage thoughts about the future and the prospect of continuing life alone.

Spouses expressed how using the intervention website had made them aware of the importance of pursuing their own interests and maintaining a separate, ongoing life not defined by illness. This was described as crucial for managing the strain that the illness placed on the relationship and for restoring energy levels, enabling spouses to continue to provide care and support to the patient. Patients actively supported their spouse by encouraging them to pursue their own social life and provide as much freedom as possible, as a couple reflected:

*Spouse: “Recuperation, how to restore energy levels and the value of staying in touch with friends. That struck me when I read it [on the intervention website], I found one text in particular to be spot on, I’ve been neglecting this. I need to reach out and go for walks with friends, otherwise it all shrinks and shrinks. I need a little more self-care.”*

*Patient: “And I’ve really encouraged you to do that. You shouldn’t have to be tied to me the whole time.” (Couple 2.* Husband, 75 years old, caring for his 74-year-old wife)

## Discussion

### Main findings of the study

This study shows that this family caregiver-targeted web-based psychoeducational intervention influenced couples’ experiences of sharing everyday life while facing life-threatening illness. Spouses’ new knowledge and understanding transferred to the patients, which enabled couples to manage the impact of illness. The intervention encouraged spouse-patient conversations that helped couples maintain mutuality. Spouses’ access to the intervention supported couples in balancing the tension between previous and new relational roles. These findings align with Andershed and Ternestedt’s theoretical framework,^31^ which guided the intervention development. While the framework’s concepts of knowing, being, and doing overlap, the findings show that spouses gained knowledge (knowing), initiated conversations (being), and balanced roles (doing). It is therefore reasonable to assume the intervention enhanced involvement “in the light” among participating spouses. Although these concepts were not specifically searched for in the analysis, they were apparent in the findings. The intervention directly addressed, for example, initiating conversations and dual roles as a caregiver, contributing to these findings. Moreover, using Interpretive description as an analytic approach provided valuable insights into couples’ experiences through interpretation. It is important to acknowledge that the authors’ comprehensive knowledge of the theoretical framework may have biased the results in favor of the three concepts. However, this was carefully considered and discussed throughout the analysis process.

### What this study adds

Our findings show that this intervention provided knowledge that enhanced mutual understanding of how both spouses and patients were affected by the illness. Videos, such as “Many different emotions,” which specifically address how caregiving-related emotions can affect spouses, using a personal tone with close trustful conversations and advice, likely facilitated this recognition. This aligns with previous research^38,39^ indicating that spousal caregivers use web-based resources to manage strain caused by the caregiver role and highlights the value of interventions to improve dyadic coping through increased mutual awareness of family caregivers’ needs.^40^ Through understanding, spouses adjusted their reactions toward the patient, making sharing everyday life easier for the couples. Psychoeducational interventions aim to provide education and knowledge, promoting changes in family caregivers’ understanding and actions. This can make family caregivers more confident in their interactions with the patient.^41^ Importantly, such interventions can also legitimize the caregiver role, aligning with palliative care strategies that advocate for greater involvement of family caregivers in research.^23^

In our study, couples reported that the intervention initiated conversations that helped them maintain mutuality. It offered practical advice on important topics to discuss, including guidance through videos on how family caregivers could initiate conversations about difficult subjects and relate to the other person’s perspective, which reasonably facilitated these conversations. Previous research emphasizes the role of communication in reducing spousal caregiver distress^42^ and maintaining mutuality within couples.^43,44^ Productive communication has been highlighted as particularly important for spousal caregivers to manage complex caregiving.^45^ Our findings provide further insight into how interventions can support couples in maintaining open communication.

Couples in our study struggled to balance relational roles, as everyday life largely revolved around the patient due to the illness. Spouses’ access to the intervention helped rebalance these roles by addressing the dual roles as a caregiver and spouse. The content provided practical advice for managing these roles, including videos emphasizing the importance of allowing yourself as a family caregiver to experience joy, pursue personal interests, and find ways to regain strength. Examples include the videos “Being a family member and a family caregiver” and “Being me”. Previous research shows that as health declines, patients may seek alternative ways to provide emotional support to compensate for their perceived inability to respond to their spouses’ needs.^46,47^ However, couples felt the intervention’s focus on family caregivers alone was a limitation, suggesting that patients should also have access. This underscores the need for interventions that address the mutual needs of couples facing life-threatening illness. Our study can inform future development of family caregiver support, aligning with both national^48^ and international^49^ policies promoting web-based resources for equity and accessibility in health care. Our findings may provide a means of fulfilling the responsibility to support family caregivers as emphasized in palliative care definitions and models.^50–52^

### Strengths and limitations

Strengths of this study include the use of dyadic interviewing, which provided unique narratives through couples’ interactions, allowing for a more comprehensive evaluation. However, it also had limitations. Couples were not asked to explain why they chose not to participate, though some expressed discomfort with participating as a dyad.

It should be acknowledged that our findings may have been influenced by that, among the participating couples, most family caregivers were female, and most patients had been diagnosed with cancer. Furthermore, couples who chose to participate may have been more resourceful or positive about the intervention. Efforts were made to encourage honest and balanced reflections, however, awareness of the research team’s involvement in intervention design may have influenced participants’ responses to highlight its benefits.

## Conclusions

The web-based intervention facilitated everyday life for couples through increased understanding, initiated conversations, and supported balancing the tension in relational roles. Benefits of family caregiver-targeted interventions may extend from spouse to patient, supporting them as couples. Our findings can inform future intervention development by providing insights into how an intervention like this could be beneficial. The goal should be for interventions to potentially benefit both patients and family caregivers.
